# Proton Pump Inhibitors and Fractures in Adults: A Critical Appraisal and Review of the Literature

**DOI:** 10.1155/2021/8902367

**Published:** 2021-01-15

**Authors:** Silvia Irina Briganti, Anda Mihaela Naciu, Gaia Tabacco, Roberto Cesareo, Nicola Napoli, Pierpaolo Trimboli, Marco Castellana, Silvia Manfrini, Andrea Palermo

**Affiliations:** ^1^Unit of Endocrinology and Diabetes, University Campus Bio-Medico, Rome, Italy; ^2^Unit of Metabolic Diseases, Department of Internal Medicine, S. Maria Goretti Hospital, 04100 Latina, Italy; ^3^Scienza Biomediche, Università Della Svizzera Italiana (USI), Lugano, Switzerland; ^4^Population Health Unit, National Institute of Gastroenterology “Saverio de Bellis”, Research Hospital, Bari, Italy

## Abstract

Despite the large number of patients worldwide being on proton pump inhibitors (PPIs) for acid-related gastrointestinal disorders, uncertainty remains over their long-term safety. Particularly, the potential side effects of these drugs on bone health have been evaluated in the last years. The purpose of our narrative review is to gather and discuss results of clinical studies focusing on the interactions between PPIs and fracture risk. Data generated mainly from nested case-control studies and meta-analysis suggest that long-term/high-dose PPIs users are characterized by an increased risk of fragility fractures, mainly hip fractures. However, in these studies, the PPIs-induced bone impairment is often not adjusted for different confounding variables that could potentially affect bone health, and exposure to PPIs was reported using medical prescriptions without adherence evaluation. The mechanisms of the PPI-related bone damage are still unclear, but impaired micronutrients absorption, hypergastrinemia, and increased secretion of histamine may play a role. Clinicians should pay attention when prescribing PPIs to subjects with a preexistent high risk of fractures and consider antiosteoporotic drugs to manage this additive effect on the bone. However, further studies are needed to clarify PPIs action on the bone.

## 1. Introduction

Proton pump inhibitors (PPIs) are a class of medications frequently prescribed all around the world. Esomeprazole became one of the most prescribed drugs, in consequence to the high number of diagnosis of gastrointestinal disorders as gastroesophageal reflux disease and peptic ulcers [1]. In the class of PPIs, considering the releases on the pharmaceutical market, it is possible to identify different molecules that share the common capability in reducing gastric acid secretion. PPIs act by irreversibly blocking the hydrogen/potassium adenosine triphosphatase enzyme system (the *H* + /*K* + ATPase or the gastric proton pump) of the gastric parietal cells, which make the secretion of *H*+ ions in the gastric lumen possible [2]. The PPIs are given in an inactive and lipophilic form, which reaches cell cytoplasm crossing cell membranes. In an acid environment, the inactive drug is protonated and rearranges into its active form, linking covalently and irreversibly to the gastric proton pump, deactivating it. The proton pump represents the ideal target for inhibiting acid secretion because of its leading role in creating an acid environment in the stomach; as a result, PPIs are extremely effective in reducing the pain from indigestion and heartburn. However, stomach acids are necessary to absorb calcium, proteins, vitamin B_12_, drugs, and other nutrients. Therefore, in conditions of prolonged hypochlorhydria, their absorption can result in impairment.

The aim of our narrative review is to gather and discuss results of clinical studies focusing on the interactions between PPIs and fracture risk.

## 2. Methods

We searched for articles published on PubMed, EMBASE, and the Cochrane Library from inception up to December 2020, according to PRISMA guidelines [3, 4] to identify published original articles and meta-analyses concerning PPIs and bone health. In particular, we searched for articles that investigated the effects of PPIs on fracture risk. The following keywords were searched: proton pump inhibitors or PPIs, lansoprazole, omeprazole, rabeprazole, pantoprazole, esomeprazole, osteoporosis, bone turnover markers, BMD, fracture, falls, osteoblast, and osteoclast.

We searched for articles published in English and those involving human participants. At the same time, we hand-searched the reference lists of the retrieved articles or meta-analyses to identify additional relevant studies. To minimize differences, studies were included if they met the following criteria: (1) those that were cohort studies, case-control studies, cross-sectional studies, randomized controlled studies, or meta-analysis, (2) the exposure of interest was PPIs use and the outcomes were fractures. Exclusion criteria included nonprimary research, review articles, lack of an outcome related to the relationship between PPIs and bone health, abstract-only publications, or non-English language publications. Additional exclusion criteria for full-texts included pediatric population, case reports, and specific conditions recognized to negatively affect bone health, such as stroke, Alzheimer's, hemodialysis, and kidney transplant. Two investigators (AP and AMN) independently searched papers, screened titles, and abstracts of the retrieved articles, reviewed the full-texts, and selected articles for their inclusion. In case of disagreement, definitive reporting was achieved by mutual consensus.

**Figure 1:**
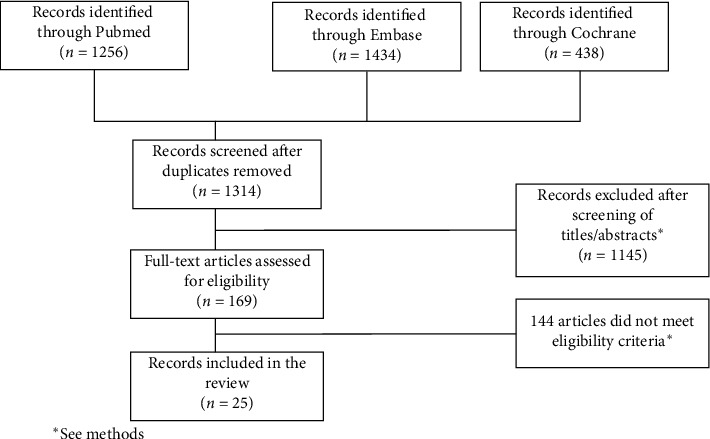
Study selection process.

A total of 1,256 studies were found through PubMed, 1,434 through Embase, and 438 through Cochrane, and after removal of all duplicates, 1,314 articles were analyzed for title and abstract; 1,145 records were excluded (reviews, letters, commentaries, posters, case reports; interventions not in humans, studies including pediatric population, or assessing specific conditions recognized to negatively affect the bone health). The remaining 169 studies were retrieved in full-text, and 25 articles corresponding to 18 case-control studies, 6 meta-analyses, and one retrospective evaluation using an aggregated knowledge-enhanced database (Administration Adverse Event Reporting System Data Mining Set) were finally included in the review (Figure 1). No additional study was retrieved from references of included studies.

## 3. Potential Mechanisms of PPIs Induced Fracture Risk

**Figure 2:**
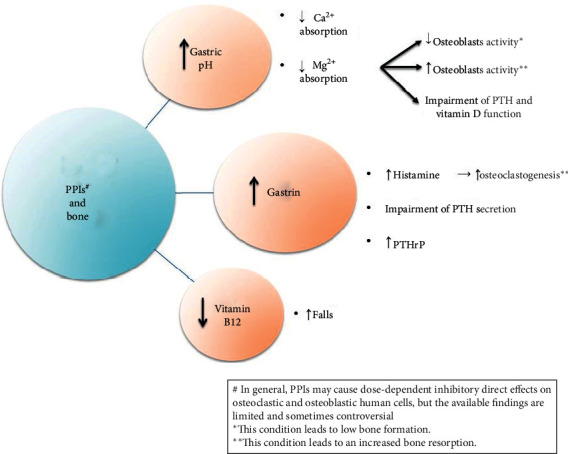
Potential mechanisms of PPIs induced fracture risk.

PPIs, histamine antagonists, and other antiacid medications have improved the quality of life of patients affected by many gastrointestinal disorders. It has been demonstrated that a chronic use of PPIs is associated with potential adverse drug events, such as hypomagnesaemia, interstitial nephritis, and iron and vitamin B12 malabsorption [5]. During the past 10 years, the relationship between PPIs and bone health (Figure 2) has received attention from many investigators. In particular, PPIs seem to be associated with an increased risk of osteoporotic fractures [6], with a primary potential mechanism involving the physiological effects of chronic acid suppression on calcium, magnesium, and parathyroid hormone (PTH) metabolism [5]. The effect of PPI on bone cells has not been widely described, and available findings are limited and sometimes controversial [7–11]. In general, PPI may cause dose-dependent inhibitory effects on osteoclastic and osteoblastic human cells leading to a kind of low bone turnover syndrome. Therefore, PPI-related bone fragility might be determined by the impairment of the repair mechanisms for bone microfractures that occur daily. However, PPIs use does not appear to determine significant bone quality impairment [12].

### 3.1. Calcium Absorption

Calcium carbonate, one of the most common dairy calcium salts, is maximally absorbed when administrated together with a meal. Indeed, it is routinely suggested to take calcium carbonate supplements with food [13]. The acidic gastric environment stimulates the release of ionized calcium from insoluble calcium salts. Despite calcium salts precipitate in the small intestine, some calcium ions can be found in solution too. In human subjects with physiological acid secretion, insoluble calcium salts, mainly if taken after a meal, are very well absorbed as soluble calcium salts [14]. Otherwise, in hypo or achlorhydric patients, the absorption of insoluble calcium salts such as calcium carbonate, in fasting conditions, virtually does not occur, while soluble calcium salts such as calcium citrate are still absorbed normally [15]. PPIs, enhancing gastric pH, could interfere with calcium absorption. It can lead to a negative calcium balance with consequent secondary hyperparathyroidism and increased bone loss [5, 16, 17]. Chonan et al. in an animal in vivo evaluation showed that rats on omeprazole therapy presented calcium malabsorption, and the reduction of intestinal pH with dietary lactate prevented the calcium absorption in rats fed with omeprazole [18]. In humans, O'Connell et al. confirmed that, in a population of ≥65 years old women, a daily omeprazole dose of 20 mg significantly reduced fasting absorption of calcium carbonate after only 7 days of treatment [19]. Moreover, impaired calcium absorption has been observed in patients after gastrectomy [20]. However, few studies in this field show controversial results.

Serfaty-Lacrosniere et al. reported that, among young healthy subjects, omeprazole therapy did not reduce the absorption of calcium contained in milk and cheese, probably in consequence to the “meal effect” and to the high bioavailability of calcium salts in these foods [21]. Hansen et al. confirmed that, in a population of postmenopausal women in therapy with omeprazole, after one month of treatment, there were not any observed differences in the absorption of calcium ingested with a meal [22]. Finally, Wright et al. did not find any differences between calcium absorption or urinary calcium excretion on 12 healthy volunteers, with or without a three-day therapy with omeprazole 20 mg twice per day enrolled in a placebo-controlled, double-blind, crossover study [23].

#### 3.1.1. Summary

PPIs increase gastric pH, and most of the evidences seem to confirm that they may negatively affect the calcium absorption. The controversial results of some studies can be related to short-term or low-dose PPIs therapy.

### 3.2. Hypergastrinemia

More recently, research has focused its attention on PPIs' relationship with osteoclasts and PTH, the most important calcium-regulating hormone, responsible for maintaining stable calcium concentrations [5]. PTH stimulates bone resorption, increases renal tubular calcium resorption and, through the action of calcitriol, enhances the upper intestine calcium reabsorption [15]. PPIs inhibit HCl secretion in the gastric lumen causing a significant increase in gastric pH, a reduction in somatostatin release from mucosal D cells located in the gastric antrum, and in consequence, an increase in serum gastrin levels. Somatostatin and gastrin act in balance one with the other, so that antiacid medications indirectly cause hypergastrinemia by suppressing somatostatin release. PPIs are excreted by the kidney, so that their compensatory hypergastrinemia appears higher in patients with impaired kidney function in consequence of a reduced drug clearance [24]. Patients in therapy with PPIs present a 2–6-fold increase in serum gastrin in comparison with control subjects [25], especially during the first months of treatment, with a progressive plateau [26]. Enhanced levels of gastrin, responsible for an enterochromaffin-like cells (ECL cells) hyperplasia, induce histamine secretion. Histamine links to receptors localized on mature and precursor osteoclasts promote osteoclastogenesis and bone resorption [27]. However, to date, there are no in vivo human studies that have investigated the impact of hypergastrinemia-induced histamine secretion on bone health. According to this concept, an observational study suggested that the blockage of type 1 histamine receptors on osteoclasts with an H1 receptor antagonist may reduce the risk of hip fractures (adjusted OR: 0.86, 95% CI 0.79–0.93), regardless of PPIs use [28]. Moreover, hypergastrinemia has been shown to have a stimulatory effect on the parathyroid glands. In vivo animal studies demonstrated that omeprazole-induced hypergastrinemia was associated with hyperparathyroidism, with an increase in parathyroid gland volume and weight, and consequent osteopenia [29]. Similar outcomes were obtained in chickens after 5 weeks of omeprazole administration with a resulting hyperplasia and hypertrophy of the parathyroid glands, an increased PTH gene expression and a reduction in femur density [30]. As a proof that all these modifications recognize hypergastrinemia as the major cause, a direct infusion of gastrin increased the weight of the parathyroid glands and reproduced the effect of omeprazole on PTH gene expression in the same animal model [31]. There are only a few in vivo human studies on this topic, and the findings are contrasting [32]. An earlier publication demonstrated that, in subjects with primary hyperparathyroidism, PTH does not affect gastrin levels and that chronic moderate hypercalcemia does not raise serum fasting gastrin, at least in clinical conditions [33]. Instead, Zaniewski and colleagues demonstrated that chronic hypercalcemia of either parathyroid or nonparathyroid origin may elevate serum gastrin concentrations [34]. Moreover, a few evidence support the hypothesis that gastrin may positively regulate parathyroid hormone-related peptide (PTHrP) expression [35].

#### 3.2.1. Summary

PPI-induced hypergastrinemia stimulates histamine and might enhance PTH secretion that may promote osteoclastogenesis and bone resorption. Few in vivo animal and human studies seem to confirm these findings.

### 3.3. Hypomagnesaemia

PPIs use may be associated with hypomagnesaemia [5]. In particular, a dose response between the PPIs use and development of hypomagnesaemia has been described in literature [36]. Magnesium is an oligoelement involved in bone metabolism both directly and indirectly. Several studies in vitro [37] and in human [38] and animal [39] models have demonstrated that magnesium deficiency reduces osteoblastic activity, detected through the measurement of alkaline phosphatase and osteocalcin, and proliferation. An increase in the number of osteoclasts in conditions of low magnesium levels has also been observed [40]. The imbalance between osteoblastic and osteoclastic differentiation should be likely determined by an enhanced activity of the nitric oxide synthase promoted in the condition of hypomagnesaemia [41]. Furthermore, nitric oxide results involved in bone marrow vasculature modifications, responsible for low magnesium induced-endothelial dysfunction, contribute to the decline of bone mass [42]. In addition to these mechanisms, magnesium deficiency affects bone homeostasis interfering with PTH and vitamin D function [43]. Studies in vitro demonstrated that acute changes in magnesium levels affects PTH secretion (an acute decrease in magnesium concentration increases PTH secretion and vice-versa) [44]. Moreover, in vivo studies have shown that hypomagnesaemia impairs PTH secretion and makes target organs refractory to PTH. PTH signaling involves the adenylate cyclase enzyme, which requires Mg as a cofactor [45]. Similarly, 25-hydroxycholecalciferol-1-hydroxylase requires Mg to promote the hydroxylation of vitamin D intermediates, so that magnesium deficiency could reduce the activity of these two different enzymatic pathways [46]. Finally, hypomagnesaemia promotes oxidative stress, inducing the production of free radicals and cytokines such as TNF*α*, IL-1s, and IL-6 [47], which stimulate osteoclast activity and differentiation [48], contributing to the imbalance between osteoblasts and osteoclasts [49].

#### 3.3.1. Summary

PPI-induced hypomagnesaemia may determine the imbalance between osteoblasts and osteoclasts, interferes with the hydroxylation of vitamin D intermediates, and makes target organs (renal and bone) resistant to PTH action.

### 3.4. Increased Falls

Another proposed mechanism that could explain the increase in fracture risk with chronic PPIs use is the increased rate of falls. Indeed, PPIs determine vitamin B12 deficiency in consequence to malabsorption [50]. One prospective cohort study found that patients treated with PPIs were more at risk to have deficient vitamin B12 levels compared to those not treated with a PPIs. Furthermore, in this study, patients treated with PPIs reported numbness of the feet and visual disturbances, a side effect of vitamin B 12 deficiency, which can certainly contribute to an increased risk of falling [51]. A case-control study enrolling 64,399 elderly Swedish patients hospitalized for a fall evidenced that the use of acid suppression drugs caused an increased risk of falling [52]. In addition, Lewis et al. in a large prospective cohort of postmenopausal elderly women found that a continuative therapy with PPIs for 1 year or more was associated with an increased risk of falls and fracture-related hospitalizations. This study also found that patients treated with long-term PPIs had an increased risk of self-reported falling (adjusted OR: 1.51, 95% CI 1.00–2.27) [53].

#### 3.4.1. Summary

PPI-induced vitamin B12 deficiency may lead to an increased rate of falls with the consequent increased risk of fractures.

### 3.5. PPIs and BMD

Some authors have investigated the relationship between PPIs and BMD changes, but literature offers contrasting results.

A prospective evaluation showed that after 12 months, subjects using PPIs, in particular, esomeprazole, had lower femur neck and total hip BMD T scores [54]. In a large nested case-control study published in 2010, PPIs were associated with a marginal effect on 3-year BMD change at the hip (0.74%, 95% CI (0.01–1.51)) but not at other skeletal sites [55]. In accordance with these previous studies, other cross-sectional evaluations showed that PPIs users had higher rates of osteoporosis at hip than controls [56, 57].

However, the association between PPIs use and hip fracture is probably related to factors partially independent of low bone mass. In fact, some cross-sectional [53, 58] and longitudinal evaluations [58–61] showed that PPIs use does not seem to be associated with BMD loss. These findings are supported by two recent meta-analyses [62, 63].

#### 3.5.1. Summary

PPIs users might not be associated with an increased femoral bone loss, but findings are not conclusive.

### 3.6. PPIs and Risk of Fractures

The characteristics of the included studies are provided in Table 1. Studies regarding risk of fracture and PPIs included in this review were all case-control studies and have been published from 2006 to 2020. No randomized controlled study was found. A great heterogeneity exists among the studies. Different PPIs were used, and some studies did not specify the type and dosage. Heterogeneity between studies was also noted for age, and the sample size ranged from 261 to 192,028 PPIs users. There was a high variability in the exclusion of comorbidities that can affect bone health. In addition, 8 studies (40% of the included studies) did not adjust the fracture risk for major confounding factors, such as BMD. The minimum length of PPIs exposure was at least one prescription. Great differences in the follow-up time were further sources of heterogeneity.

Focusing on hip fractures, in 2006, Yang et al. conducted a case-control study showing that long-term PPIs therapy exposes users to an increased risk of hip fracture. In particular, the risk significantly increased among patients on long-term and high-dose PPIs (OR: 2.65, 95% CI 1.80–3.90) [64]. In accordance with previous findings, in 2014, Cea Soriano et al. highlighted that PPIs' use modestly increased risk of hip fracture (OR: 1.09, 95% CI 1.01–1.17) [68]. When compared to H2-receptor antagonists (H2RA), the initiation of PPIs seems to be associated with a higher risk of hip fracture (HR: 1.27, 95% CI 1.09–1.48) [78].

However, evidences reported an increased risk of fractures also at nonfemoral sites [69, 81]. In particular, Vestergaard et al. investigated the effects of one-year therapy with PPIs on fracture risk. They demonstrated that the use of PPIs not only increased the risk of hip fracture (OR: 1.45, 95% CI 1.28–1.65) but also of fractures at spine (OR: 1.60, 95% CI 1.25–2.04) and at any skeletal sites (OR: 1.18, 95% CI 1.12–1.43) [65]. In 2010, Gray performed a nested case-control study on 130,487 women with a follow-up of 7.8 years. After multivariate analysis, the risk for the spine (HR: 1.47, 95% CI 1.18–1.82), lower arm/wrist (HR: 1.26, 95% CI 1.05–1.51), or all site (HR: 1.25, 95% CI 1.15–1.36) fractures resulted increased, but this finding was not observed in hip (HR: 1.00, 95% CI 0.71–1.40) [55].

The PPIs-induced fracture risk seems to be confirmed also after adjusting the analysis for multiple risk factors, including femoral neck bone density and use of bisphosphonates (BP) (HR: 1.40, 95% CI 1.11–1.77). [66]. The impairment of bone health has been shown in both Caucasian [70] and Asian cohorts [67, 75, 77]. In particular, Lee et al. described the increase risk of hip fractures in PPIs users/BP nonusers in comparison with PPIs/BP nonusers in a large cohort of Korean subjects (OR: 1.34, 95% CI 1.24–1.44). However, the risk was not dose and/or drug-dependent in PPIs users/BP nonusers [67]. Very recently, in another cohort of Korean population, Park et al. evaluated 8,903 elderly women suffering osteoporotic fractures and 44,515 matched controls without fractures between 2009 and 2015. Short- and long-term PPIs use showed a positive association with the risk of osteoporotic fracture (OR: 1.3, 95% CI 1.09–1.56 and OR: 1.31, 95% CI 1.23–1.38, respectively), and cumulative dose did not show any influence over fracture risk [75].

PPIs use would negatively affect the fracture risk also in men. In particular, Adams et al. confirmed the relationship between the dose and duration of PPIs use and hip fracture risk in 7,000 nonHispanic Caucasian men (long use—OR: 1.23, 95% CI 1.02–1.48; recent use—OR: 1.22, 95% CI 1.02–1.47) [71].

In the last years, some meta-analyses have investigated the impact of PPIs use on the development of fragility fractures. The authors concluded that PPIs use may be associated with an increased risk of hip [62, 82, 83] and any-site fracture [63, 84, 85].

PPIs seem also to increase the risk of refractures in elderly population. In particular, Brozek et al. analyzed retrospectively the impact of PPIs use on the risk of hip refracture in a population of 31,668 patients who suffered hip fracture between July 2008 and December 2010 excluding antiosteoporotic users. The authors demonstrated that PPIs therapy increased the risk for subsequent hip fracture (OR: 1.58, 95% CI 1.25–2.00), in particular in 70–84-year-old men; however, these findings were not confirmed in case of low dosage (OR: 1.35, 95% CI 0.99–1.82) and short-term therapy (OR: 1.14, 95% CI 0.77–1.69) [80].

In contrast, some authors showed that PPIs do not affect the risk of fracture, regardless of ethnicity, duration of the therapy, and the dose, absolute or cumulative [73, 74, 79]. In particular, in a very recent retrospective investigation, Hoff et al. demonstrated in a Norwegian population of 15,017 women and 13,241 men, aged 50–85 years, that PPIs therapy did not expose to an increased fracture risk in either women (HR: 0.80, 95% CI 0.65–0.98) or men (HR: 1.00, 95% CI 0.69–1.45) [76].

#### 3.6.1. Summary

Most evidences support that long-term high-dosage PPIs users are characterized by the increased risk of fractures.

### 3.7. Efficacy of Antiresorptive Agents and PPIs

Treatment of osteoporosis is aimed to prevent fragility fractures and to stabilize or increase bone mineral density [86]. It has been demonstrated that PPIs users are more likely to subsequently have osteoporosis therapy than nonusers [72], but it is not clear if PPIs can affect the efficacy of antiosteoporotic therapy in preventing fractures. A small prospective investigation [87] and a post hoc analysis of 3 RCT trials [88] reported that, regardless of PPIs concomitant use, risedronate significantly reduced the risk of new vertebral fractures compared with placebo [88] and increased the BMD [87, 88]. Same findings have been found for the use of teriparatide [89].

As shown by Alhambra et al. and Abrahamsen et al., PPIs use might predict the occurrence of fractures independently of compliance and persistence to BP therapy [90, 91].

Moreover, a meta-analysis published by Yang et al. found that the overall fracture risk of BP and PPIs users versus only BP users was increased [92].

In contrast, there is only one prospective study conducted on osteoporotic patients using concomitant PPIs that has shown a greater increase in lumbar BMD after one year of treatment with alendronate in comparison with alfacalcidiol. However, although the design of study is adequate, the small sample size affects the data interpretation [93].

#### 3.7.1. Summary

Poor and incomplete data do not allow providing clear clinical information on the interaction between PPIs and BPs.

## 4. Conclusions

Data generated mainly from nested case-control studies and meta-analyses suggest that long-term/high-dose PPIs users are characterized by an increased risk of fragility fractures, mainly hip fractures. However, in these studies, the PPIs-induced bone impairment is often not adjusted for different confounding variables that could potentially affect bone health, and exposure to PPIs was reported using medical prescriptions without adherence evaluation. Impairment of calcium, magnesium, and vitamin B absorbance, hypergastrinemia, an increased secretion of histamine, play an important role in determining the increased fracture risk, although the pathophysiological mechanisms of the PPIs-related bone damage are still unclear.

Physicians should carefully evaluate the risk of fractures in long-term high-dose PPIs users and suggest adequate calcium/vitamin D supplementation. Clinicians should pay attention when prescribing PPIs to subjects with a preexistent high risk of fractures, and they may consider antiosteoporotic drugs to manage this additive effect on the bone. Antiosteoporotic drugs still remain the best option for the management of PPIs users with high risk of fracture.

However, further studies are needed to clarify PPIs' action on the bone and to estimate the real risk of fracture.

## Figures and Tables

**Table 1:** Main characteristics of studies regarding PPIs and risk of fractures.

Author [Ref]	Year	Study design	Aim of the study	Study population	Intervention	Main findings	Main limitations
Yang et al. [64]	2006	Nested case-control study	To explore the association between PPIs therapy and risk of hip fracture.	192,028 PPIs users, 187,686 users of H2RA, and 1.4 million acid suppression nonusers older than 50 yrs	PPIs use >1 year before hip fracture. Daily dosages were divided in high and low dosage.	After adjustment for some potential confounding factors (comorbidities and drugs), long-term PPIs therapy, particularly at high doses, is associated with an increased risk of hip fracture (OR: 2.65, 95% CI 1.80–3.90)	Authors did not adjust the results for major confounding factors that can affect bone health such as BMD.
							The study was not designed to define the mechanisms between PPIs therapy and the risk of fracture.
							Data about calcium supplementation were not available.

Vestergaard et al. [65]	2006	Case-control study	To evaluate the effect of PPIs, H2RA, and other types of antacid drugs on fracture risk	124,655 subjects with any fracture sustained during the year 2000 and 373,962 controls	PPIs, H2RA, and other types of antacid drugs	For less than 1 year since the last use of PPIs, an increased risk of fracture was registered (OR: 1.18, 95% CI 1.12–1.43).	For more than 1 year since the last use, the risk disappeared for PPIs
							Authors did not adjust the results for major confounding factors that can affect bone health. Information regarding BMD, smoking, physical activity, the use of calcium or vitamin D supplements, sun exposure, or risk of falling were not available.

Gray [55]	2010	Nested case-control study	To evaluate the association of PPIs use with BMD changes and fractures	161,806 postmenopausal women (50–79 years): 3,396 PPI users	PPIs use with a mean follow-up of 7.8 years.	After adjustment for major potential confounding factors (comorbidities and drugs), PPIs use was not related to risk for hip fracture but was related to clinical spine (HR: 1.47, 95% CI 1.18–1.82), lower wrist/arm (HR: 1.26, 95% CI 1.05–1.51), and total fractures (HR: 1.25, 95% CI 1.15–1.36). A marginal effect on 3 years BMD change at hip was associated with PPIs use.	Self-reported fractures.
							Lack of information on dose of PPIs drugs.

Fraser et al. [66]	2013	Nested case-control study	To investigate if PPIs use increased the risk of fragility fractures	9,423 participants in the Canadian Multicenter Osteoporosis Study: 261 PPIs users	PPIs use with a follow-up of 10 years	After adjustment for major potential confounders factors, PPIs use was associated with a shorter time to first fragility fracture (HR: 1.40, 95 % CI 1.11–1.77).	PPIs drug use data only for years 0, 5, and 10 of the study (lack of information between these time points).
							PPIs dose was not accounted in this study.

Lee et al. [67]	2013	Nested case-control study	To evaluate the relation between PPIs and risk of hip fractures and to explore the interaction between PPIs and BPs	24,710 cases of incident hip fractures (1,282 PPIs users) and 98,642 controls > 65 years old identified from January 2005 to June 2006	PPIs use: at least one prescription of PPI between 2005 and the index date.	Elderly subjects using PPIs presented an increased risk of hip fracture (OR: 1.34, 95% CI 1.24–1.44). Subjects using BP and with a current use or high cumulative dose of PPIs had a higher risk of hip fractures.	Current medication was reported using prescriptions (also subjects with only 1 prescription were enrolled).
							Authors did not rule out all the condition that can affect bone health such as the utilization of drugs, low BMI, alcohol consumption, and smoking.
Cea Soriano L. et al. [68]	2014	Nested case-control study	To evaluate the association between hip fracture risk and use of PPIs	10,958 subjects (PPI users 2,699) with hip fractures and 20,000 controls aged 40–89 years.	PPIs use with a mean follow-up of 5.5 years	Use of medium-high dose of PPIs (in particular, omeprazole) was associated with a modest increased risk of hip fracture after adjustment for major potential confounders (including the presence of osteoporosis) (high dose OR: 1.31, 95% CI 1.06–1.61).	Information about, family history, diet, and physical activity were not available.

Ding et al. [69]	2014	Nested case-control study	To evaluate the relationship between PPIs use/adherence and fracture risk among	1,604 PPIs users and 23,672 nonusers (>65 years old)	PPIs use with a follow-up of 5 years	Subjects with higher adherence at PPIs therapy were associated with grater fracture risk after adjustment for some potential confounders (HR: 1.27, 95% CI 1.12–1.43).	Authors did not rule out some major conditions that can affect bone health. Information about BMD, calcium and vitamin D intake, physical activity, and alcohol use were not available.
							Adherence to treatment and fractures were identified by using administrative claims data.
							By using diagnostic codes for incident fractures (without validation through imaging or procedure coding), some false-positive fractures might be included.
							1 yr baseline period was used to exclude prior fractures and PPIs usage.

Moberg et al. [70]	2014	Nested case-control study	To investigate risk factors at baseline for first fracture in postmenopausal women during long-term follow-up	6,917 women >50 years old: 121 PPIs users	PPIs use with a mean follow-up of 14.4 years	After adjustment for major potential confounding factors, the use of PPIs was associated with a doubled risk of fracture (OR: 2.53, 95% CI 1.28–4.99).	Lack of information on duration of PPIs use.
							The baseline information was self-reported.
							Clinical vertebral fractures were undiagnosed, and the true number of fractures might be higher than reported.

Adams et al. [71]	2014	Nested case-control study	To estimate the association between PPIs use and hip fracture	6,774 men with a hip fracture (896 used omeprazole and 694 used pantoprazole) and 6,774 controls aged ≥45 years old	PPIs use (omeprazole and pantoprazole) with a follow-up of 10 years	Most adherent, long-term (OR: 1.23, 95% CI 1.02–1.48) or most recent (OR: 1.22, 95% CI 1.02–1.47) PPIs users (men) presented an increased risk of hip fracture	Authors did not adjust the results for major confounding factors that can affect bone health, such as BMD.
							Pharmacy records are limited to dispensed prescriptions.

Van der Hoorn et al. [72]	2015	Nested case-control study	To evaluate the effect of dose and type of PPIs use on subsequent use of osteoporosis drugs and fractures in older Australian women	4,432 women >77 years old (2,328 PPIs users)	PPIs use with an average follow-up of 6.6 yrs	PPIs use is associated with an increased risk of the subsequent use of osteoporosis medication (HR: 1.28, 95% CI 1.13–1.44) and fractures (HR: 1.29, 95% CI 1.08–1.55) in older women	Residual confounding factors such as vitamin B12 deficiency, celiac disease, and lower dose of corticosteroids were not included in this study.
							Lack of information on compliance with PPIs use.
							The fractures outcome was based only on hospital admission data.
							Self-reported chronic conditions.
							Exposure to PPIs was reported using prescriptions (also subjects with only 1 prescription were enrolled).

Lai et al. [73]	2018	Nested case-control study	To explore the relationship between PPIs use and hip fracture	7,208 subjects with newly diagnosed hip fracture (PPIs users 2,083) and 7,208 controls without fracture (PPIs users 1,776), >65 years old	PPIs use	After adjustment for some confounding factors, no significant association was found between PPIs use and the risk of hip fracture (OR: 0.96, 95% CI 0.76–1.22)	Authors did not adjust the results for major confounding factors that can affect bone health, such as BMD. Data on other pharmacological treatment was not available.

Harding et al. [74]	2018	Nested case-control study	To evaluate if the use of PPIs is associated with an increased fracture risk	4,438 participants aged ≥65 years old (408 PPIs users)	PPIs use with a mean follow-up of 6.1 years	After adjustment for some potential confounding factors, older adults using PPIs presented no increased fracture risk (HR: 1.14, 95% CI 0.91–1.42).	Authors did not adjust the results for major confounding factors that can affect bone health, such as BMD. Lack of information about alcohol use, calcium and vitamin D intake, and the family history of fractures.
							Diagnosis codes were used to ascertain fractures.

Park et al. [75]	2020	Nested case-control study	To compare the risk of osteoporotic fracture between PPIs users and H2RA-only users	8,903 subjects with new osteoporotic fractures (4,301 PPIs users) and 44,515 matched controls >66 years old	PPIs and H2RA use with a mean follow-up of 1.7 years	After adjustment for major confounding factors, long-term and recent PPIs uses have been associated with an increased risk of osteoporotic fractures (OR: 1.31, 95% CI: 1.23–1.38) compared with H2RA-only use.	High rate of nonparticipating Korean women.
							Osteoporotic fractures have been ascertained without reviewing medical records and imaging data.
							Not all traumatic fractures have been ruled out.
							The effects of PPIs in subjects with a history of fractures have not been evaluated because patients with previous fractures were excluded.

Hoff et al. [76]	2020	Nested case-control study	To examine the association between the use of PPIs and risk of fractures	28,258 subjects (4,490 PPIs users) aged 50–85 years.	Average PPIs use of 3.8 yrs	Use of PPIs was not associated with an increased risk of fractures even after adjustment for some confounding factors in both women (OR: 0.80, 95% CI 0.65–0.98) and men (OR: 1.00, 95% CI 0.69–1.45)	Authors did not adjust the results for major confounding factors that can affect bone health. Lack of information about BMD, alcohol use, calcium and vitamin D intake, and the family history of fractures.
							Only hip and forearm fractures have been registered.

Park et al. [77]	2020	Nested case-control study	To investigate the risk of osteoporotic fractures associated with PPI use compared to exclusive H2RA use in Korean population	59,240 subjects with osteoporotic fractures (23,311 PPIs users) with a mean age of 64.8 ± 8.0 and 296,200 controls (103,742 PPIs users)	Median PPIs use of 30 days	Use of PPIs was associated with an increased risk of osteoporotic fractures even after adjustment for confounding factors (OR: 1.11, 95% CI 1.08–1.13) compared to H2RA users	Residual confounding factors that can affect bone health.
							Lack of information about BMD.
							The osteoporotic fractures were defined using the diagnostic codes of claims data

Wei et al. [78]	2020	Propensity score-matched cohort study	To compare the risk of incident hip fracture between PPIs and H2RA initiators	50,265 PPIs and 50,265 H2RA users >50 years old	Average PPIs use of 3.7 yrs	The initiation of PPIs was associated with a higher risk of hip fracture than the initiation of H2RA (HR: 1.27, 95% CI 1.09–1.48).	Subjects with higher PPIs prescriptions had high risk for hip fracture (OR: 1.67, 95% CI 1.33–2.10)
							Authors did not adjust the results for major confounding factors that can affect bone health.
							Lack of information about BMD and the family history of fractures.

Reyes et al. [79]	2013	Retrospective case-control study	To assess if the use of PPIs is associated with an increased risk of hip fracture in a Mediterranean area	358 cases (202 PPIs users) matched with 698 controls (312 PPIs users), >50 years old	PPIs use in the 5 years before the hip fracture	The use of PPIs was not associated with an increased risk of hip fracture after adjusting for major risk factors (including the presence of osteoporosis) (OR 1.17, 95% CI 0.77–1.79)	Lack of information about alcohol use, calcium and vitamin D intake, and the family history of fractures.
							Exposure to PPIs was reported using prescriptions (also subjects with only 1 prescription were enrolled).

Brozek et al. [80]	2019	Retrospective cohort study	To examine the association of PPIs use with hip fracture	31,668 Austrian patients ≥50 years with a first hip fracture (13,262 PPIs users)	PPIs use with a median follow-up of 1.54 years	Low-dose PPIs use is not associated with the increased risk of subsequent hip fractures (OR: 1.35, 95% CI 0.99–1.82), especially in women.	Information on comorbidities and comedications except for antiosteoporotic drugs was not available.
							Lack of information on BMD, calcium and vitamin D intake, physical activity, BMI, alcohol consumption, and smoking.

PPIs, proton pump inhibitors; H2RA, H2-receptor antagonists; BMD, bone mineral density; BP, bisphosphonates.

## Data Availability

The data supporting this review are from previously reported studies and datasets, which have been cited.
